# Reactive Lymphoid Hyperplasia of the liver in a patient with colon cancer: report of two cases

**DOI:** 10.1186/1471-230X-6-25

**Published:** 2006-09-12

**Authors:** Hiroki Takahashi, Hirozumi Sawai, Yoichi Matsuo, Hitoshi Funahashi, Mikinori Satoh, Yuji Okada, Hiroshi Inagaki, Hiromitsu Takeyama, Tadao Manabe

**Affiliations:** 1 Department of Gastroenterological Surgery, Nagoya City University Graduate School of Medical Sciences, Kawasumi 1, Mizuho-cho, Mizuho-ku, Nagoya, Japan; 2 Department of Pathology, Nagoya City University Graduate School of Medical Sciences, Kawasumi 1, Mizuho-cho, Mizuho-ku, Nagoya, Japan

## Abstract

**Background:**

Reactive lymphoid hyperplasia (RLH) of the liver is very rarely reported, and we encountered two cases of RLH of the liver in a patient with colon cancer.

**Case presentation:**

In the first case, a 77-year-old woman was admitted for the surgical removal of a ascending colon cancer. A hepatic tumor in the left lobe was concurrently revealed by computed tomography (CT), and magnetic resonance imaging (MRI). The appearance suggested liver metastasis. Right hemicolectomy and partial hepatectomy were performed. On histopathological examination, lymphoid follicles with germinal centers were seen in the tumor-like lesion, and remarkable lymphoid infiltration with germinal centers was seen in the portal area around the nodule. Immunohistochemical studies revealed polyclonality of infiltrating lymphocyte. Consequently, this nodular lesion was diagnosed as RLH of the liver. In the second case, a 64-year-old woman who had a radical right hemicolectomy for stage II ascending colon cancer 10 years ago was admitted with dysuria. A hepatic tumor in the left lobe was concurrently revealed by CT and MRI, suggesting hepatocellular carcinoma. A left lateral segmentectomy was performed. Microscopically, this lesion revealed the almost same findings as the first case, so this nodular lesion was diagnosed as RLH of the liver.

**Conclusion:**

Our two cases were the first report of RLH of the liver accompanying colon cancer. Because there are a very few cases, so it is not clear whether the malignancies were involved in the onset of RLH. But we believe that new factors involved in the onset mechanism of RLH may be identified by carefully monitoring the clinical course of our two patients.

## Background

Reactive lymphoid hyperplasia (RLH) is reported to occur in the gastrointestinal tract [1], skin [2], lung [3], thyroid [4], orbit [5], and pancreas [6]; however, it is very rarely reported in the liver. To the best of our knowledge, only 12 cases with RLH of the liver have so far been reported in the English literature [7-15]. RLH is thought to represent an immune-mediated reactive phenomenon, and very few cases may arise in association with malignant tumor. But, there are no reports mentioned about the relationship between RLH and malignant tumor. Our two cases were the first report of RLH of the liver accompanying colon cancer. So we report two cases of RLH of the liver accompanying colon cancer and discuss its morphological features.

## Case presentation

### Case 1

**Figure 1:**
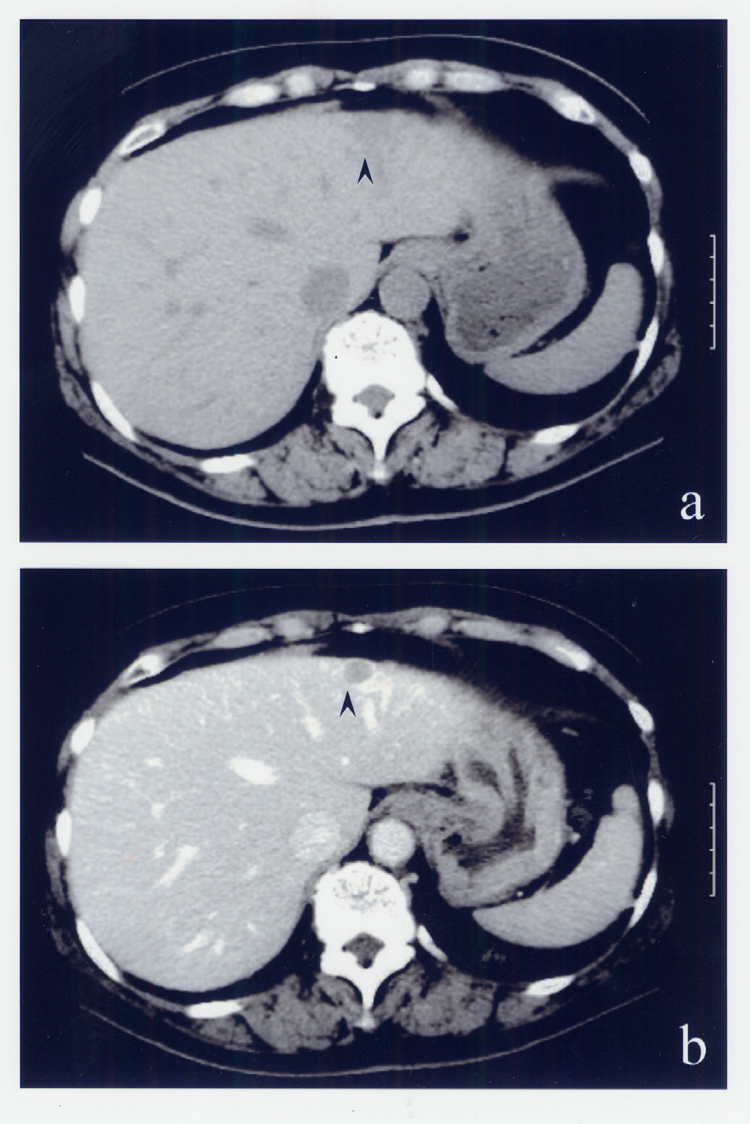
a) Abdominal CT shows the 1.5-cm diameter low-density mass in size in the S3. b) This lesion was slightly enhanced, and its periphery was noticeably enhanced on equilibrium phase.

**Figure 2:**
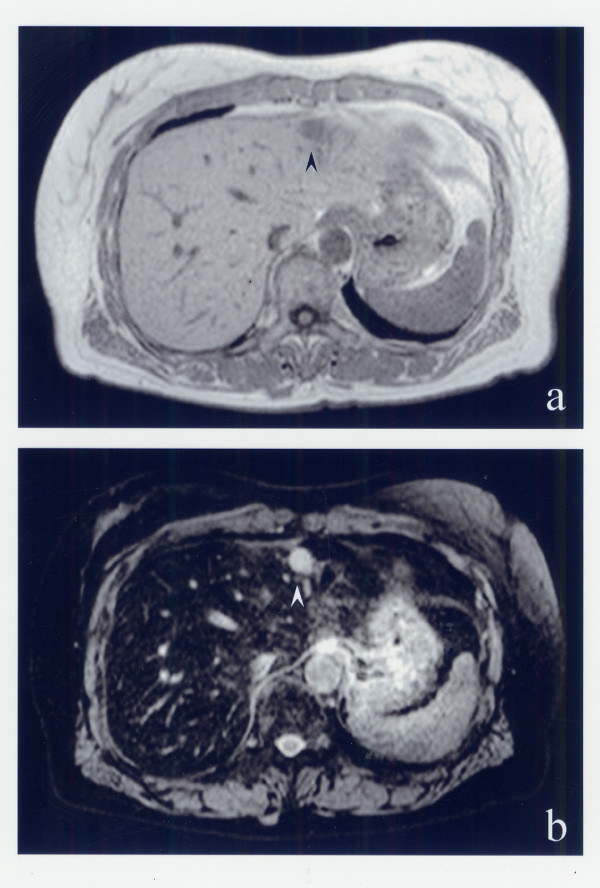
a) MRI showed a low-intensity lesion on the T1-weighted image. b) The border of the lesion was clearly visualized on the T2-weighted image with SPIO.

A 77-year-old woman was admitted to our hospital with right upper abdominal pain in December 2004. Barium enema and colonoscopy revealed an ulcerative tumor in the ascending colon, which was diagnosed as moderately differentiated adenocarcinoma on biopsy. Physical examination showed no remarkable abnormalities. Neither hepatomegaly nor splenomegaly were apparent. Serum hepatitis B surface antigen and hepatitis C antibody were negative, and she had no history of alcohol intake or blood transfusion. Laboratory data on admission including liver function tests were unremarkable. Carcinoembryonic antigen (CEA), carbohydrate antigen 19-9 (CA19-9), and α-fetoprotein (AFP) were within normal ranges. Abdominal ultrasonography showed a hypoechoic, 1.5-cm diameter mass in the left hepatic lobe (S3). Computed tomography (CT) revealed a low-density area in the same area, the interior of which enhanced equally. The outline of the mass was particularly enhanced in equilibrium phase (Fig. 1). Magnetic resonance imaging (MRI) demonstrated a slightly low-intensity lesion on T1-weighted images; this was high-Intensity on T2-weighted images (Fig. 2). Furthermore, superparamagnetic iron oxide (SPIO) uptake was not detected on T2-weighted images. Consequently, as metastatic liver tumor could not be definitively excluded, the patient underwent right hemicolectomy and S3 partial hepatectomy on 1 November 2004.

**Figure 3:**
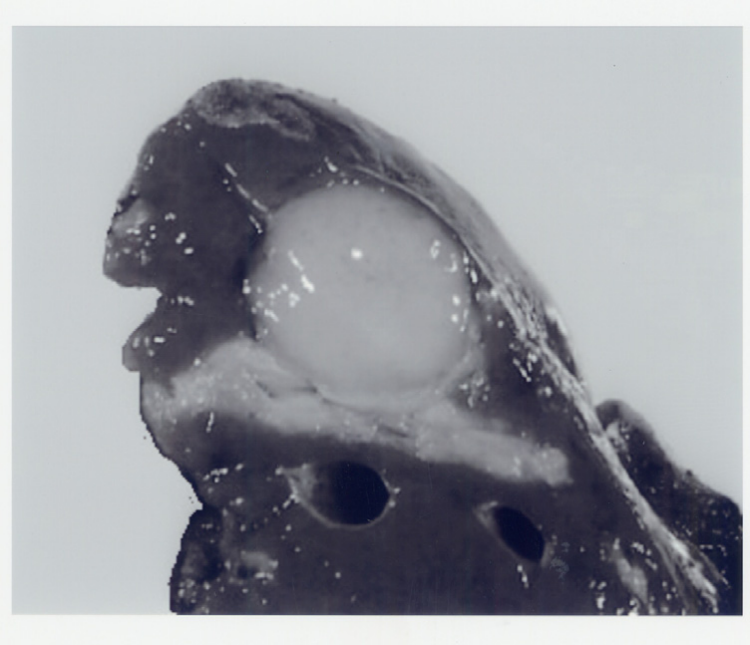
A cut section of the resected liver showed a well-circumscribed, yellowish-white mass in S3.

**Figure 4:**
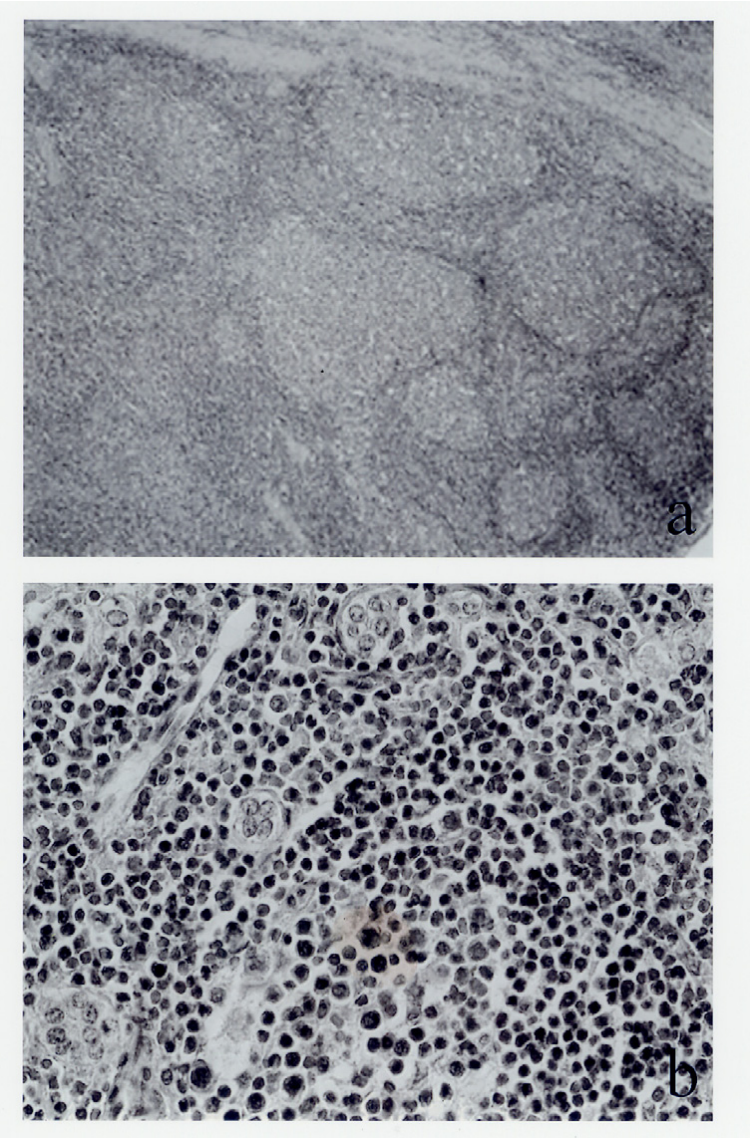
Many lymphoid follicles with germinal centers were seen in the tumor-like hepatic lesion (a: ×40, b: ×400), HE staining.

An ulcerated tumor with sharply demarcation was present in the ascending colon and histopathological examination revealed moderately differentiated adenocarcinoma. No lymph node metastasis was detected. Macroscopically, cut section of the resected liver showed a well-circumscribed, encapsulated, yellowish-white mass in S3 of the liver. It measured 1.5 cm in diameter (Fig. 3). The tumor appeared relatively soft and elastic and was composed of many lymphoid follicles with large germinal centers containing small polymorphic lymphocytes (Fig. 4). Many plasma cells surrounded mature lymphoid aggregates. Furthermore, remarkable lymphoid infiltration with germinal center formation was seen in the portal area around the nodule. However, the hepatic parenchyma distant from this nodule was normal and lymphoid infiltration was not detected in the portal area. No evidence of adenocarcinoma metastasis and no cytologic atypia of the germinal centers or interfollicular areas were detected. Immunohistochemical studies revealed the germinal centers to be composed of CD-20-positive and Bcl-2-negative lymphocytes. Furthermore, lymphocytes surrounding the germinal centers and interfollicular areas were positive for CD-3 and Bcl-2. Plasma cells were positive for both κ and λ light chains at an equal frequency (data not shown). These findings revealed the polyclonal origins of the infiltrating lymphocytes. Consequently, this nodular lesion was diagnosed as RLH of the liver. No recurrence has occurred during 20 months of postoperative follow up.

### Case 2

**Figure 5:**
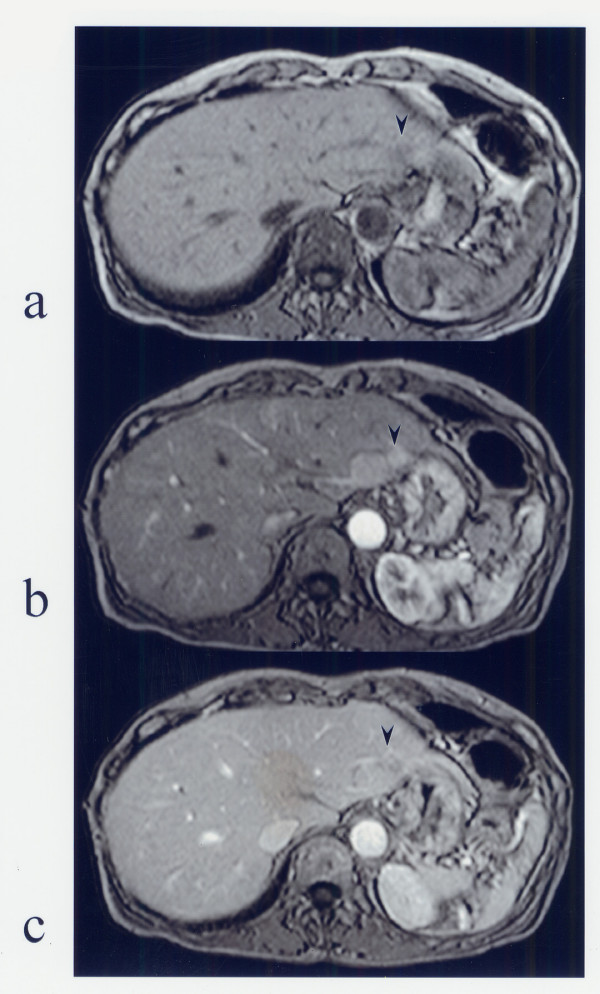
Dynamic MRI. a) Plain MRI showed the 1.0-cm diameter low-intensity mass in size in the S2. b) This nodular lesion was enhanced at an early phase. c) At a late phase, this lesion was showed as low-intensity mass than surrounding liver tissue.

A 64-year-old woman who had a radical right hemicolectomy for stage II ascending colon cancer 10 years ago was admitted to our hospital with dysuria in July 2005. A hepatic tumor in the left lobe of her liver was concurrently revealed by CT scan. Serum hepatitis B surface antigen and hepatitis C antibody were negative, and she had no history of alcohol intake or blood transfusion. Laboratory data on admission including liver function tests were unremarkable. CEA, CA19-9, and AFP were within normal ranges. Abdominal ultrasonography showed a hypoechoic, 9 × 7 mm diameter mass in the left hepatic lobe (S2). Plain CT examination revealed a low-density area, the mass was enhanced by the contrast medium in early phase. At a late phase, the mass was showed as a low-density area. MRI demonstrated a slightly low-intensity lesion on T1-weighted images and a high-Intensity on T2-weighted images. SPIO uptake was not detected on T2-weighted images. At an early phase, Dynamic MRI revealed low-intensity mass in S2 (Fig. 5a). At a late phase, the mass was showed as a low-intensity area (Fig. 5b). Consequently, this nodular lesion was diagnosed hepatocellular carcinoma rather than liver metastasis, the patient was carried out left lateral segmentectomy on 7 September 2005.

**Figure 6:**
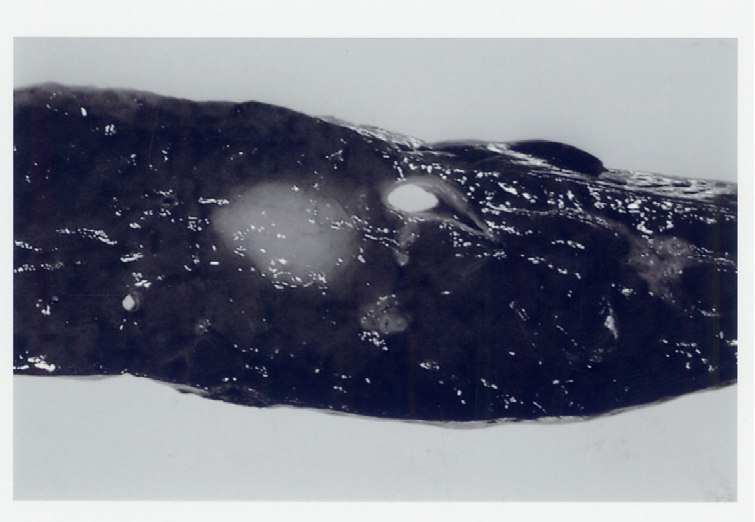
A cut section of the resected liver showed a well-circumscribed, whitish mass in S2.

Macroscopically, the resected liver had smooth surface and a normal appearance, cut section of the resected liver showed a well-circumscribed, not encapsulated, whitish mass in S2 of the liver (Fig. 6). It measured 1.0 cm in diameter. Microscopically, this lesion revealed the almost same findings as *Case 1*, so this nodular lesion was diagnosed as RLH of the liver, too. No recurrence has occurred during 10 months of postoperative follow up.

## Discussion

Reactive lymphoid hyperplasia is recognized as a region that is well demarcated and characterized by hyperplastic lymphoid follicles with reactive germinal centers, which consist of polymorphic and polyclonal cell populations and aggregations of mature lymphocytes, plasma cells, and other types of inflammatory cells [12]. In the liver, these reactive lesions have been variously termed RLH, nodular lymphoid lesions [14], and pseudolymphoma. These lesions are very rare, and they appear clinically to indicate the same disease. We decided to use the term "RLH of the liver" because this most closely reflects the pathological features of the disease. However, the term "pseudolymphoma" is problematic because it indicates a low-grade type of MALT lymphoma. Since malignant transformation is reported in this type of MALT lymphoma, surgical resection is indicated. Therefore, even when pseudolymphoma is diagnosed preoperatively, some patients have undergone resective surgery [13]. In contrast, as RLH is not considered to have malignant potential, therapeutic strategies are totally different from those for low-grade MALT-type lymphoma. We therefore believe it is necessary to eliminate the ambiguous term "pseudolymphoma" and clearly differentiate RLH from low-grade MALT-type lymphoma. Some past studies have also advocated this approach to classification, and Sharifi and colleagues [14] have proposed clear diagnostic criteria. Immunohistological and genetic tests to ascertain whether infiltrating lymphocytes are monoclonal or polyclonal enable differentiation of RLH from low-grade MALT-type lymphoma.

By previously reported cases, in terms of imaging findings, RLH of the liver is depicted as a hypoechoic lesion on ultrasound and as a low-density lesion on plain CT, but contrast CT findings vary. Both MRI T1-low and T2-high intensity images yield the same results, and angiography shows hypervascularity. In our patients, results of preoperative diagnostic imaging conformed to this pattern; i.e. low-echo and plain CT demonstrated a low-density lesion, while contrast CT showed mild enhancement. Moreover, on equilibrium phase of contrast CT in the first patient, the area around RLH was depicted as a high-density area, possibly indicating a capsule. The present study was the first to conduct contrast SPIO-enhanced MRI in hepatic RLH. No SPIO uptake into the mass occurred, and the boundary with the normal liver was very clear. The further discussion of such rare cases based on the imaging findings including SPIO-enhanced MRI will provide more information about this rare entity. But, at this point in time, it is very difficult to completely rule out malignancies such as HCC, metastatic liver tumors, or malignant lymphomas based solely on diagnostic imaging findings. Ultimately, preoperative biopsy is therefore an essential diagnostic tool.

While the cause of RLH has not been clarified, most patients are middle-aged or elderly women. In terms of underlying diseases, various chronic inflammatory diseases, such as chronic hepatitis, Sjogren's syndrome, chronic thyroiditis, and primary biliary cirrhosis, have been confirmed. Hence, autoimmune mechanisms are most likely involved. In our cases, the patients were a middle-aged woman and lymphoid infiltration was observed in the portal area around the nodule, suggesting a similar mechanism. However, lymphoid infiltration was confined to the area around the nodule, the rest of the hepatic parenchyma was normal, and hepatitis virus markers were negative, suggesting that this lesion was local rather than diffuse. Furthermore, as systemic immunologic abnormalities were not detected, our cases did not appear to be associated with systemic autoimmune disease. Including the present patients, five cases of hepatic RLH accompanying malignancies have been reported [8,11,15], some of which had asynchronous onset, but the present study is the first to report RLH of the liver accompanying colon cancer. Because there are a very few cases, so it is not clear whether the malignancies were involved in the onset of RLH. Furthermore, characteristic imaging findings or morphological features in these patients were not detected. But we believe that new factors involved in the onset mechanism of RLH may be identified by carefully monitoring the clinical course of our two patients.

## Conclusion

In summary, we report two cases of RLH of the liver in a patient with colon cancer. RLH of the liver is extremely rare and it is very difficult to think of RLH of the liver as differential diagnosis.

## Abbreviations

RLH: Reactive lymphoid hyperplasia, MALT: Mucosa-associated lymphoid tissue, US: ultrasonography, CT: Computed tomography, MRI: Magnetic resonance imaging, SPIO: Superparamagnetic iron oxide

## Competing interests

The author(s) declare that they have no competing interests.

## Authors' contributions

HT, MS, YO, and HT carried out the clinical examination and operation. HT, HF, HS, and HI performed pathological analysis. HT and YM participated in the design of the study. TM conceived of the study, and participated in its design and coordination. All authors read and approved the final manuscript.

## Pre-publication history

The pre-publication history for this paper can be accessed here:



## Figures and Tables

**Table 1:** Reported cases of reactive lymphoid hyperplasia of the liver.

No.	age	sex	segment	Size(cm)	CT	MRI	US	Angio	association	Author/Year
1	69	F	Rt post lobe	1.7*1.0*0.9	plain: low not enhanced	-	-	-	7 yeas after RCC ope.	Pantanowitz/2001
2	47	F	S7	1.7	plain: low enhanced	T1- low T2-high	hypoechoic hyperechoic rim	hypervascular	Hashimoto's thyroiditis	Nagano/1999
3	67	F	Lt lobe	2	plain: low sl. enhanced	-	hypoechoic	hypervascular	-	Tanizawa/1996
4	52	F	Rt lobe	0.4	-	-	-	-	primaly biliary cirrhousis	Sharifi/1999
5	56	F	Lt lobe	1.5	-	-	-	-	primaly biliary cirrhousis CREST synd.	Sharifi/1999
6	56	M	-	0.7	-	-	-	-	chronic diverticular	Sharifi/1999
7	15	F	-	-	-	-	-	-	primary immunodeficiency synd.	Snover/1981
8	49	F	Rt post lobe	2	Tumor lesion (+)	T1-low T2-high	tumor lesion (+)	hypervascular	Sjögren's synd.	Okubo/2001
9	42	F	S6, S3	S3:1.1 S6:1.6	low density Enhance-CT:iso	-	hypoechoic	tumor stain(+)	chronic hepatitis B, IFN-α	Ohtsu/1994
10	72	M	S3	2.1*1.4	Plain: no tumor Early: high, late: iso-low	T1-low T2-high	hypoechoic	failure	chronic hepatitis C early gastric cancer	Kim/1997
11	66	F	Rt lobe	1.0*1.0	plain: low not enhanced	-	hypoechoic	tumor stain(+)	DM	Katayanagi/1994
12	85	F	Rt lobe	1.4*0.8*1.0 0.7*0.8*0.7	-	-	-	-	gastric cancer	Grouls/1984
**Our cases**										

1	77	F	S3	1.5	plain: low, enhance sl.(+)	T1-low T2-high	hypoechoic	-	colon cancer	
2	64	F	S2	0.9*0.7	Plain: low, enhance-CT Early:high, late:low	T1-low T2-high	hypoechoic	tumor stain(-)	10 years after colon cancer	
